# The Duty of the State

**Published:** 1888-03-03

**Authors:** 


### THE DUTY OF THE STATE.

The whole theory of State care of the weak and dependent seems to me to be justifiable only so far as we are sure that we are replacing incapacity by something more than shelter, vicious experiences by something more than colourless experiences or even non-experiences, and bad habits by good or better habits. Education is chiefly the registering in the nervous system of preferred experiences. The country boy—Jack-of-all-trades—has often more capacity than his city representative, merely because his experiences have been so much more varied and extensive. The boy who grows up outside an institution generally leads a more varied, and therefore (physiologically speaking) more instructive life. Life in an institution should be vigorous, busy, nervous, full of "doing things," playing things, anything except inactivity and sleepy quiet.—*Professor W. T. Sedgwick.*

